# Insight Under Scrutiny in the Court of Protection: A Case Law Survey

**DOI:** 10.3389/fpsyt.2020.560329

**Published:** 2020-09-11

**Authors:** Sándor Gurbai, Emily Fitton, Wayne Martin

**Affiliations:** ^1^The Essex Autonomy Project, School of Philosophy and Art History, University of Essex, Colchester, United Kingdom; ^2^Human Rights Centre, University of Essex, Colchester, United Kingdom; ^3^Faculty of Special Needs Education, Institute for Disability and Social Participation, ELTE Eötvös Loránd University, Budapest, Hungary

**Keywords:** insight, mental capacity, capacity assessment, Court of Protection (England and Wales), conceptual geography

## Abstract

The concept of patient insight, of a patient’s self-awareness of illness or impairment (and related issues), plays a significant role in clinical discourse and clinical practice. But what role does it play in the legal process, particularly when a patient’s decision-making capacity (or “mental capacity”) is in question? We report on a survey of 412 published judgments from the Court of Protection of England and Wales, published between 2007 and 2018. We found that the notion of patient insight played a role in 53 cases (13% of the total). We use a variety of techniques to provide a systematic profile of these “insight cases.” We provide a demographic profile of the patients whose insight is discussed (focusing on gender, age and diagnosis) and a professional profile of the expert witnesses who raise the issue of insight. We then deploy the technique of “logical geography” to map the meaning of the term insight and the inferential patterns in which reports of patient insight are embedded. We point out that the published insight cases never explicitly define “insight,” and that they include no reference to structured instruments or scales for the assessment of insight. We show that the concept of insight, as used in the Court of Protection, is not synonymous with the concept of agreement with a diagnosis of illness; this is at most one of a range of meanings that the concept carries. We show that, despite the fact that the presence or absence of insight is not itself a legal criterion for mental capacity, insightlessness does play a role, and sometimes a decisive role, in shaping findings as regards the presence or absence of mental capacity. Finally, we assess the extent to which expert testimony in the insight cases conforms to the insight-related recommendation of the recent NICE Guideline on *Decision-Making and Mental Capacity*.

## Introduction

In 2018, the UK government commissioned an independent review of the Mental Health Act (England and Wales) —the so-called *Wessely Review*. The final report of the review included a call for:

Research into how the legal test of decision-making capacity in the MCA can be translated into practice. This should include: … the relationship with clinical concepts such as “insight”; … [(1): 226].

This paper is the first of a series of papers in which we present the results of an extended, multipronged, multidisciplinary research initiative addressing the relationship between insight and decision-making capacity. The principal aim of this first paper is descriptive. Focusing on the published judgments of the Court of Protection (CoP) (England and Wales), we map the current usage of the concept of patient insight in legal proceedings that pertain to findings of decision-making (in)capacity. We assess current usage against a recently published guidance standard.

Insight is a widely used concept in clinical discourse and clinical practice and it also plays an important role in those CoP proceedings where the mental capacity of the person concerned (P) is questioned. However, the concept of patient insight plays no explicit statutory role in England and Wales. The term makes no appearance at all in the Mental Capacity Act 2005 (MCA) or the Mental Health Act 1983 (MHA), nor does it feature meaningfully either in the MHA Code of Practice 2015 (2) or in the MCA Code of Practice 2007 (3). This statutory silence regarding the concept of insight leaves judges with considerable leeway in determining whether and how to incorporate considerations about patient insight into legal proceedings.

The use of the concept of insight in CoP proceedings raises a number of questions. How is insight defined in CoP cases? How are insight scales and scores used in these cases as evidence to show whether P has insight or lacks insight? Is insight used as a binary concept? How are the concepts of insight and mental capacity used in relation to each other? What are the socio-demographic variables in CoP proceedings that address insight, and how should these variations be understood?

To address these questions, we proceed as follows. We begin (§2) by reviewing the existing academic literature on the place of the concept of insight in legal proceedings, examining both the literature that has focused on the role of insight in mental health tribunals and the more limited literature that focuses on the role of insight where decision-making capacity is the principal legal concern. We then (§3) present the methodology we relied upon in our survey. In §4 we provide a demographic profile of the patients whose insight is discussed, with particular attention to the differential patterns with respect to gender and insight. In §5 we consider the professional profiles of the expert witnesses who discuss insight. We then turn to the inferential patterns in which reports of patient insight are embedded, focusing first (§6) on patterns pertaining to the meaning of the insight construct and then (§7) on the patterns in which insight is related to mental capacity. We conclude (§8) by considering the extent to which expert testimony in the CoP conforms to the insight-related recommendation of the National Institute for Health and Care Excellence (NICE) *Guideline on Decision-Making and Mental Capacity* (2018) (4).

One final preliminary: This paper presupposes familiarity with the concept of “mental capacity” (or “decision-making capacity”) and its place in law, as also with the role of the CoP in making legal determinations when capacity is disputed. Readers are referred to the excellent recent article by Ruck Keene et al. (5), for a broader survey of these matters.

## Existing Research Regarding Insight in Legal Proceedings

The concept and phenomenon of insight, its measurement and its correlation with other variables, has been the subject of academic research around the world. However, the bulk of published research has either addressed principally clinical issues or else has focused on the legal implications of impaired insight in mental health tribunals and in other legal procedures governed by Mental Health Acts. As a consequence, research on the interface between insight and the law has tended to focus on the role of insight and insightlessness in decisions to “section,” to discharge from section, to resort to coercive psychiatric interventions, etc. What has been less systematically studied (albeit with one or two notable exceptions, of which more below) has been the use of the concept of insight in legal proceedings in which a person’s *decision-making capacity* is the central issue.

In reviewing this literature, we begin with an overview of studies of the role of insight in mental health tribunals and related Mental Health Act proceedings. One pioneering work in this area was Peay (6), which analyzed how the Mental Health Act 1983 was put into practice in England and Wales. Peay reported that when it came to deciding on the patient’s discharge, Mental Health Review Tribunals placed considerable emphasis on the question of whether the patient had insight or not and focused particularly on the question of whether the patient understood the need to take their medication [(6): 143]. Peay’s finding was supported by Perkins et al., who found that, among tribunal members, insight was the single most consistently discussed symptom of mental illness [(7): 124].

Diesfeld (8) reached a similar set of conclusions concerning the work of tribunals in New Zealand, while Freckelton (9) found that tribunal decision-making in Australia regularly departed from legislatively prescribed considerations in relation to release, relying on substitute criteria such as “lack of insight.”

Both Diesfeld and Freckelton were critical of the role that findings regarding insight play in tribunal proceedings. Diesfeld complained that the ‘liberty interests of detained patients may be compromised when tribunals utilize ill-defined, extra-legislative criteria in determining whether to begin, or extend, psychiatric detention’ [(8): 380]. According to Diesfield, the use of insight is an example of such a criterion. For his part, Freckelton argued that reliance on findings regarding insight ‘can be both unfair and ill-conceived. First, it can constitute a de facto decision that certain matters must be proved before a patient can be released; second, it can involve reference to terms that are unhelpfully imprecise; third, it can introduce extra-legislative considerations which may not be justifiable’ [(9): 53].

Kress (10) argued that the time had come for the concept of insight to have a formal place in mental health law, particularly in the law governing coerced treatment and civil commitment, but also in the criminal law. Kress held that “because lack of insight negates central knowledge essential to an informed, autonomous decision, intervention by government may well be justified on the ground that non-autonomous behavior does not deserve the same rights and protections as autonomous action.” [(10): 268].

Diesfeld and McKenna (11) reported that the term insight had been constantly in use before the mental health review tribunals in New Zealand, but that (i) the concept of insight was not defined, (ii) none of the decisions referred to a specific insight assessment test and (iii) none of the decisions referred to relevant research on interpreting insight. Diesfeld and McKenna also noted that “in some cases, there is an implication that a person’s insight can be located on a *scale or continuum*” [(11): 22-23].

Diesfeld and Sjöström (12) reviewed 25 decisions from mental health review proceedings in the Australian state of Victoria. They argued that the use of evidence concerning insight was problematic for three reasons, namely (i) there was little clarification of the meaning of insight, (ii) when insight was used in relation to compliance, the logic was often unclear, and (iii) there were frequent allusions to an implicit and undefined scale of insight. The authors also argued that the concept of insight was particularly relevant in navigating the sometimes conflicting goals of law and medicine and in bridging the divide between legal and clinical discourses. [(12): 86-98]

Turning to the smaller body of literature on insight and mental capacity, one key paper is Allen, 2009 (13). Allen argued that “[i]f the law is to recognize a role for insight, its meaning and relationship with capacity requires more thought and greater clarity” [(13): 169]. The most important precedent for the present study is Case (14), which reported on use of the term “insight” in CoP judgments from 2007 to 2015, and discussed three cases in some detail. Our own study goes beyond Case’s in (i) having a broader scope, (ii) distinguishing “everyday” from “technical” uses of “insight” in CoP judgments, (iii) undertaking a logical mapping of “insight,” and (iv) evaluating appeals to insight against a recently published guideline.

The most recent literature concerning insight and the law has come in response to a Supreme Court ruling in the Australian state of Victoria (*PBU & NJE v Mental Health Tribunal* [2018] VSC 564). Responding to the case, Scott and Prowacki (15) argued that “many clinicians would not accept the proposition that a person who has no insight into his or her serious mental illness, has at the same time, decision-making capacity to refuse evidence-based treatment for that serious mental illness” [(15): 7]. Invoking the findings of Trauer and Sacks (16) and Lincoln, Lüllmann and Rief (17), they also argue that ‘while poor treatment adherence mediates the relationship between insight and outcome, there also appears to be a direct association between insight and outcome [(15): 5].

Responding to the same case, Freckelton (18) reiterated some of his earlier concerns, arguing that “there is a risk that … extra-legislative notions, such as insight and compliance, will intrude into decision-making without legislative warrant … and it can operationalize prejudices and be based on paternalistic myths and irrelevancies.” [(18): 15].

## Methodology

In conducting our survey, we relied on the “British and Irish Legal Information Institute” database (BAILII) for identifying cases in which the concept of patient insight played a role in legal proceedings in the CoP. According to BAILII, 412 CoP cases were published in total between 2007 and 2018.

We gathered two sets of data in order to be able to provide both the profile of P (that is, the person who is the focus of proceedings in the insight cases) and the profile of the insight cases themselves. Based on this approach our research focused on *P-related characteristics* and *procedure-related factors*. Our P-related characteristics were the following:

- Gender of P;
- Age of P at the time of the judgment;
- Diagnosis of P.

We had planned to gather data concerning national origin of P, but this was too infrequently reported to make for a meaningful and reliable data set.

We collected data on procedure-related factors including the following:

- List of mentions of insight;
- Object of insight (i.e., insight into what?);
- Expert(s) involved;
- Who mentioned insight;
- Link between insight and related concepts including diagnosis and mental capacity.

Interrogating the BAILII database for occurrences of the search-term “insight” in judgments of the CoP (England and Wales) between 2007 and 2018, we found 89 cases and a total of 208 uses of the term.

However not all of these ‘hits’ were relevant to our aims. The term “insight” has an ordinary use in everyday discourse in addition to its technical or quasi-technical meaning in the discourse surrounding health and care. For example, one judge expressed his appreciation for two expert witnesses in the following terms:

Both Mr Entwistle and Mr Rees prepared detailed written submissions on the issues for each hearing. I am extremely grateful to both of them for the clarity of their submissions and the insight which each has brought to the issues. (*The Public Guardian v DA & Ors* [2018] EWCOP 26, para 16)

In our preliminary sift of the data set, we accordingly drew a distinction between *relevant* (i.e., technical/quasi-technical) and *irrelevant* (i.e., everyday) occurrences of the word “insight.” Occurrences were deemed to be *relevant*, for example, if insight into health condition, insight into mental illness and/or impairment, insight into care needs, insight into consequences, insight into the need for medication, insight into risk, or insight into the need for treatment was mentioned. However, when insight was used in relation to experts in order to emphasize, for example, that their contribution was insightful, or when P’s insight into other matters were the focus, we excluded those occurrences from our analysis.

Of the 208 occurrences of the term “insight” in the cases that we surveyed, we sifted out 62 instances (30%) as irrelevant, leaving a total of 146 instances (70%) as relevant. 60% of the cases in which the word “insight” occurred survived this preliminary sifting—a total of 53 cases. In the other 40% (36 cases), all uses of “insight” were deemed irrelevant to our survey. The 53 relevant cases included cases in which insight was used both in a relevant and in a non-relevant way. These 53 cases constituted 12.86% of the initial set of 412 published CoP cases. In the balance of our analysis we refer to these 53 cases as “the CoP Insight Cases,” or simply “the Insight Cases.”

## Demographic Profile of Persons Concerned

So who are the people whose insight is discussed in the CoP? In this section we provide a demographic profile. As we have seen, our survey yielded 53 Insight Cases, but in fact there were 56 individuals discussed. This was because three of the cases combined discussions of more than one individual. To provide context for our demographic profile, we used the findings of Series et al. (19) which reported on an audit of CoP applications, and used BAILII to generate a random sample (n=100) from the 412 published cases that fell within the review period, excluding judgments which involved more than one P.

### Age

**Figure 1:**
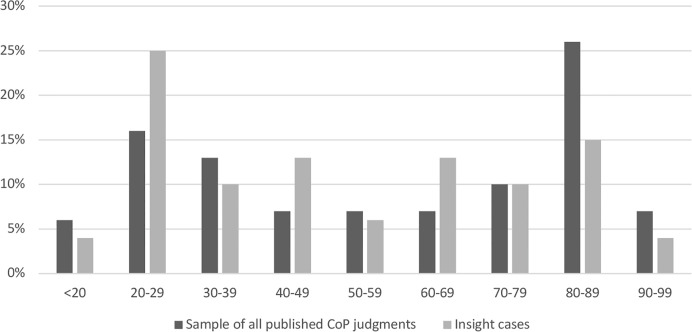
Age of P in Court of Protection (CoP) Insight Cases (n = 56) and in a random sample of all published CoP cases (n = 100); 2007–2018.

**Figure 1** provides a profile of the age of the persons who were the focus of proceedings in the CoP Insight Cases, alongside comparison data showing the age profile from our random sample from all published CoP judgments. As can be seen there, the sample from the full set of published CoP judgments skews older, with high representation from Ps aged 80 and above. The distribution of age in the Insight Cases shows two peaks, one among those under 30 (29% of the total) and a second over 80 (19% of the total).

### Gender

**Figure 2:**
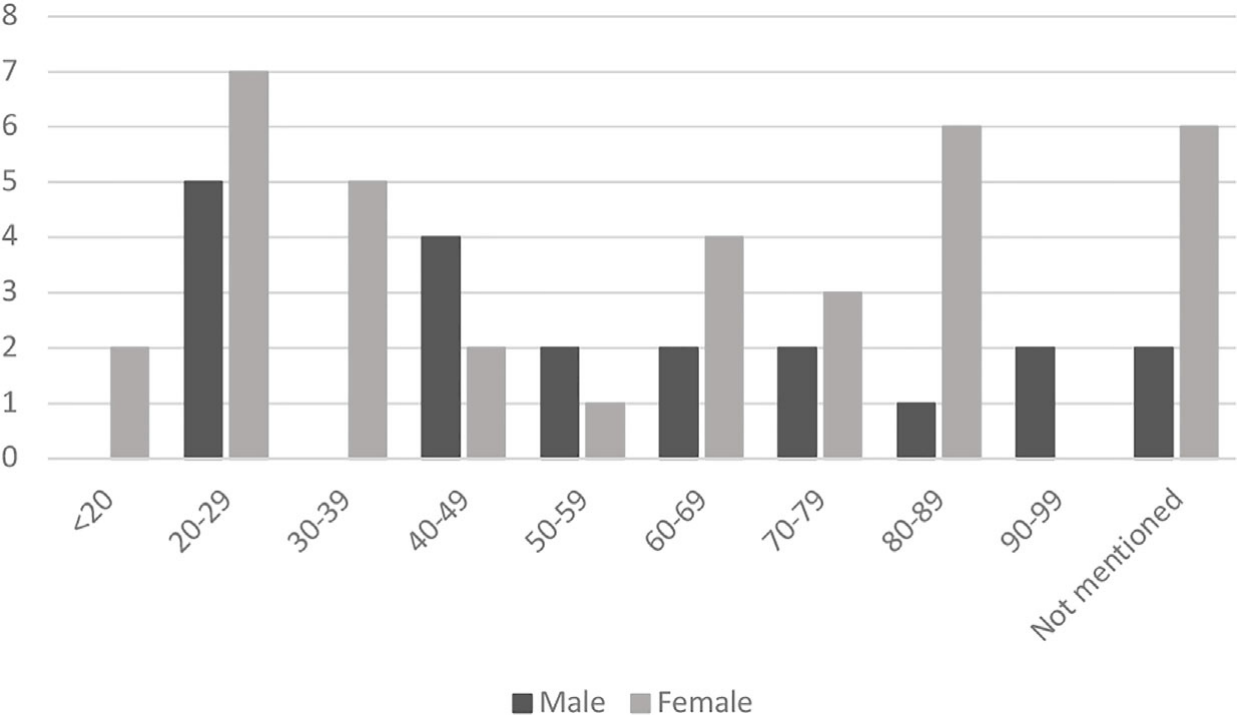
Age groups divide by gender in Court of Protection (CoP) insight cases between 2007 and 2018 (n = 56).

Series et al. (19) reported that “in most areas there was little evidence of an effect of gender on the kinds of applications received by the CoP” [(19): 38-39]. In their survey of applications, they found 118 men and 114 women [(19): 42]. We were therefore surprised to find that the gender split in the CoP Insight Cases showed a dramatic gender asymmetry, with roughly two women for every man at the focus of the proceedings. Otherwise put: almost two-thirds of the CoP Insight Cases involve women. In cases involving persons either under 40 or between 60 and 90, the gender split was nearly 3:1. **Figure 2** shows the gender split by age-group.

Care must be exercised in interpreting these findings. The overall number of cases we surveyed was not large (53 cases; 56 persons), and when divided by age group the numbers are even smaller. So no statistically significant information can be garnered from this observation. Moreover, it is important to recognize that the baseline data from Series et al. pertained to *applications* to Court. By contrast, our survey focused on *published judgments* of the Court. It is possible that the process whereby judgments are selected for publication itself introduces a gender bias. Accordingly we decided to check all 412 published cases from the CoP for the period under review, in order to determine the gender of the person who was the focus of proceedings. Note that this total includes both the CoP Insight Cases and cases that make no relevant mention of insight. Once again we were surprised: we found that the gender divide in the published CoP judgments was also approximately 2:1 (i.e., two women for every man). A number of experts with whom we have consulted have speculated about the explanation for this gender asymmetry in the published cases. We resist the temptation to enter into such speculation here. The important point for our purposes is that the gender divide in the CoP Insight Cases mirrors the gender divide in the larger pool of published CoP cases from which they are drawn.

### Diagnosis/Disability

**Figure 3:**
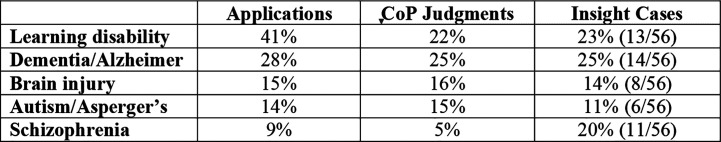
Prevalence of Diagnosis. The first column shows the prevalence of the most commonly mentioned diagnoses in applications to the Court of Protection (CoP) as reported in Series et al. [(19):43]. The second column shows the prevalence of these diagnoses in our random sample of 100 published CoP judgments. The third column shows the prevalence of these diagnoses (as percentages and as absolute values) among the 56 persons who were the focus of proceedings in the CoP Insight Cases.

Series et al. (19) provided data on the range of diagnoses and disabilities of P recorded as a stated cause of incapacity. Our research produced data on P’s diagnoses and impairments as reported both (a) in a sampling of all published CoP judgments, and (b) in the COP Insight Cases. The notable variation across these three datasets pertains to the prevalence of the diagnosis of schizophrenia, which appears in one-fifth of the CoP Insight Cases, but in only 5% of our random sample of all published CoP cases, and in only 9% of the applications surveyed by Series et al. See **Figure 3**.

### Gender, Diagnosis, Insight

**Figure 4:**
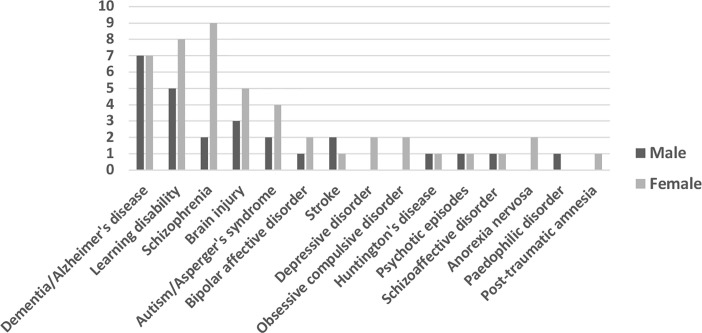
Diagnosis divide by gender in Court of Protection (CoP) Insight Cases between 2007 and 2018 (n = 56).

At the intersection of gender, diagnosis, and insight, we found at least as many women as men in all but one category of diagnosis. See **Figure 4**. Notice that there were only two men with the diagnosis of schizophrenia whose insight was discussed in the CoP Insight Cases, while we find 9 women with the same diagnosis whose insight was discussed. We would re-emphasize, however, that the absolute numbers at this level of analysis are tiny, and the background gender balance in the published CoP judgments must be taken into account.

## Professional Profile of Expert Witnesses

**Figure 5:**
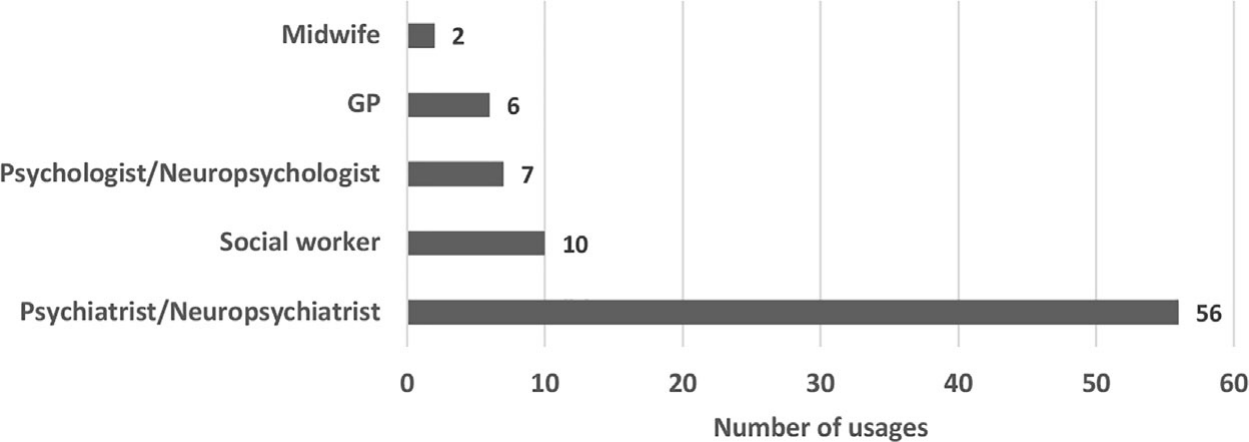
Mentions of insight by experts in Court of Protection (CoP) Insight Cases (most represented disciplines); 2007–2018.

Paula Case (14) pointed out in her study that “[w]hilst the law asserts the upper hand in the assessment of mental capacity for persons who come before the CoP, it is the discipline of psychiatry, which dominates expert witness testimony in these proceedings” [(14): 360]. Our findings confirm this in the context of the Insight Cases. We found the following:

- *Reports of insight and lack of insight were most frequently provided by psychiatrists*. See **Figure 5**. 38.4% of the total relevant usages of insight is linked to this expertise. Social workers used insight in a relevant way in 6.8% of the total usages. Psychologists and neuropsychologists mentioned insight in 4.8%, while GPs in 4.1% of the total relevant usages of insight.
- *Psychiatrists were the experts who commented on insight most frequently in the insight cases*. Out of the 53 cases where insight was mentioned in a relevant way, psychiatrists and neuropsychiatrists commented on insight in 28 cases, which represent 52.8% of the total. Social workers mentioned insight in 8 cases (15.1%), psychologists and neuropsychologists in five cases (9.4%) and GPs in two cases (3.8%).
- *Psychiatrists were the most involved experts in insight cases regardless of whether they commented on insight or not*. Psychiatrists were involved in 75.5%, social workers in 32.1%, and psychologists in 17% of the Insight Cases. For comparison, in our sampling of all published CoP judgments, testimony from psychiatrists was reported in only 34% of the judgments; social workers in 24% and psychologists in 10%.

In sum, our data clearly shows that *the leading expertise used in insight-related CoP cases was psychiatry*. Moreover, psychiatric experts were more than twice as likely to be involved in cases that address insight than they are in other CoP proceedings.

## Conceptual Geography (I): The Meaning of “Insight”

Studies of the use of the concept of insight in mental health tribunals have found that the term is often left undefined and poorly clarified [(11): 22, (11): 86-98]. Surveys of tribunal judgments in New Zealand and Australia found no tribunal judgments that relied on the results of any specific scale or test for the measurement of insight [(11): 22). Furthermore, while tribunal judgments included frequent references to insight as a scalar phenomenon, there was no evidence of reliance on any formal instrument for rating or quantifying degrees of insight [(11): 22, (11): 86-98].

The results of our survey exhibit similar phenomena in CoP rulings. Both expert witnesses and judges regularly *use* the concept of insight, but we searched in vain for any explicit *definition* or clarification of what was meant by the concept. We also found no examples of expert witnesses or judges availing themselves of insight tests or insight scales for the quantification of insight.

So what does “insight” mean in the CoP? In the absence of any clear definitions, we adopted a different approach for identifying and elaborating the specific meaning or meanings of the term as it is currently used in CoP proceedings. Following Fulford (20) we adopted a strategy of *conceptual geography*. On this method, indebted to Ryle (21) and Wittgenstein’s philosophical approach to language, one discloses the meaning or meanings of a technical term by mapping recurrent patterns of usage employed by speakers, particularly expert speakers. In this section and the one that follows we present the results of our exercise in semantic mapping. In this section we focus on patterns of usage that help identify what the term insight *means* in CoP proceedings. In the following section we focus on patterns of usage that bear on the *inferential significance* of findings of insight as regards findings of mental (in)capacity.

As we have indicated, the concepts of insight or insightlessness are nowhere defined in the published cases of the CoP. But we mapped five recurrent patterns of usage that implicitly disclosed elements of the meaning of these terms. These patterns sometimes occur independently of one another; in other cases (and even in some individual sentences), these different patterns are intertwined in a variety of ways. In what follows we name and briefly explain each of these five patterns, followed in each case by examples from the surveyed case law; emphasis in **bold print** is added throughout. In presenting these patterns we adopt a distinctive use of the double colon (::) to make clear that we are not here listing formal definitions. As we use it here, A :: B means *A is elaborated in terms of B*.

### Pattern 1

P Lacks Insight :: *P Does Not Believe/Accept/Agree That S/He Has a (Mental Health) Problem*.

Much of the published discussion of impaired insight focuses on the phenomenon in which a patient disputes their diagnosis – typically where the diagnosis is schizophrenia. This usage was well represented in the surveyed cases. Examples:

Dr O has been JB’s community psychiatrist since October 2013. She has seen her three times: October, January and 12 February. She advises that **JB lacks insight into her mental state and does not believe that she has a mental illness**. This is not uncommon with schizophrenia. (*Heart of England NHS Foundation Trust v JB* [2014] EWCOP 342, para 30)

The question of capacity is central to the jurisdiction of this court and I was satisfied that, having considered all the documents, there should be an assessment by an independent expert. Dr Hugh Series was instructed and his report is dated 27 January 2017. His opinion is that, on the balance of probability SL is suffering from paranoid schizophrenia which is a recognized mental disorder. At paragraph 7.1.5 he says that, “… schizophrenia is a condition which interferes with the process of thought, and it can undermine a person’s ability to reason and to weigh things up appropriately. It is common for a person to **lack insight into his condition and not accept that there is anything wrong. I found that SL did not agree that she had schizophrenia;** this is also reported many times in the papers in the bundle.” (*SL, Re* [2017] EWCOP 5, para 7.)

### Pattern 2

P Lacks Insight:: *P Does Not Comprehend/Understand His/Her (Mental Health) Problem*.

The second pattern of usage might be considered a close variant on the first. But rather than elaborating insight in terms of *believing or accepting* (as in the first pattern), the elaboration here concerns *comprehending or understanding a problem or impairment*. In this pattern, the problem in question was typically something other than a psychotic disorder. Examples:

I accept the expert evidence of both Doctor Ahmed and Doctor Davis. In particular, it is abundantly clear, given the cognitive deterioration which DM has sustained from chronic abuse of alcohol, that **DM has no insight or comprehension as regards his alcohol problem**, nor as to his proven inability to care adequately for himself. (*DM v Y City Council* [2017] EWCOP 13, para 16)

It was common ground that C lacked capacity to conduct the litigation and capacity to make decisions as to her health and social care needs, just as it was common ground that A and C each lacked capacity to make decisions about being locked in her bedroom at night. (Indeed, it is plain that **they have only very limited understanding of or insight into their disabilities**.) I made interim declarations to that effect… (*A Local Authority v A (A Child)& Anor* [2010] EWCOP 978, para 35)

### Pattern 3

P Lacks Insight :: *P Does Not Recognize/Understand Risk(s)*.

Although published discussions and debates about insight often focus on the awareness of illness, we found that one of the most common patterns of usage in the surveyed cases pertained not directly to awareness *of diagnosis* but specifically to the recognition or understanding *of risk*. Examples:

She was assessed as having **“very limited insight”** as to why there are significant concerns **about her behavior and the risks she was placing herself in**; she also “had limited understanding of the impact which her autism spectrum disorder has on **her ability to estimate risks** and make decisions for herself which are not going to leave her in a dangerous position.” (*Z & Ors, Re* [2016] EWCOP 4, para 40)

D concluded: “on reassessment, PH appears to have mentally deteriorated. At the time of assessment he was fixated on the notion of returning to R’s property but had limited insight into the practicalities of this idea, and I was unable to negotiate and reason with him. He did not appear to understand the information that we were discussing, nor was he processing the information in a manner that allowed for the decision-making process to be considered. **PH appeared to have no insight into the risks** that would be present in the community and he was unable to discuss with me the options for ensuring that the risks were manageable and positive.” *(PH v A Local Authority* [2011] EWCOP 1704, para 48)

### Pattern 4

P Lacks Insight :: *P Does Not Recognize/Understand Care/Support Needs*.

In a related set of cases, the “object” of insight or impaired insight pertained specifically to the person’s *need for care and support*. In some (but not all) instances this pattern was closely associated with perceptions about the value of medication (see Pattern 5). Examples:

He is at risk of self neglect because he **lacks insight into his care needs** and the need to maintain his medication. (*Bournemouth Borough Council v PS & Anor* [2015] EWCOP 39, para 10)

“**Due to [Miss V]’s lack of insight**, she is unable to understand her vulnerability when out in the community, particularly with regards to strangers approaching her and possibly asking her to get into a car, or the fact that she may get lost and be unable to find her way back. **[Miss V] showed no understanding of the support that she needs** for personal safety.” (*The Hospital Trust v V & Ors* [2017] EWCOP 20, para 42)

### Pattern 5

P Lacks Insight :: *P Fails to Recognize the Value of Treatment*.

In some academic discussions, one key factor in insight is held to be adherence with treatment. In our survey, we found few cases where the concept of insight was elaborated directly in terms of treatment-adherence; what was more common was a pattern of usage linking insight specifically with the *recognition of the need or value of treatment*. Examples:

In relation to capacity to decide where to live Dr Killaspy identified the following as relevant information[:]

(i). her lack of insight into her mental health condition and the connection between taking medication and remaining well. (*London Borough of Islington v QR* [2014] EWCOP 26, para 43)

KD [exhibited] persistent lack of insight into nature of her mental disorder and the importance for her health in complying with antipsychotic medication. (*BHCC v KD* [2016] EWCOP B2, para 16)

It should be clear from the foregoing that the terms “insight” and “insightlessness” can be used in a variety of ways. Moreover, as it is used in the Court of Protection, the concept of insight is demonstrably *not synonymous* with the concept of acceptance of or agreement with a diagnosis of illness. As we have seen, the concept has a significantly broader range of application. We return to the significance of this ambiguity for good practice in §8, below. First, however, we extend our exercise in conceptual geography to consider the matter of inferential significance.

## Conceptual Geography (II): Inferential Patterns Regarding Insight and Capacity

One of our principal aims in conducting the case law survey was to identify the role that claims about patient insight play in expert testimony and judicial rulings in the CoP. We were particularly interested in the conclusions that were drawn from evidence of insightlessness, and in the ways in which attributions of insight or insightlessness were used to support claims about decision-making capacity or incapacity. We identified eight main patterns in which these concepts occurred; emphasis in **bold print** is added throughout.

### Pattern 6

#### Bare/Atomic

In many cases, claims that a patient lacked insight were reported as “bare facts”—i.e., without evidence to support the claim of insightlessness and without any explicit indication of conclusions from that claim. In a number of these cases, claims about a patient’s insight appeared as part of a list of features of the patient’s clinical presentation. Examples:

I have also noted how **a loss of insight** appears to be a prominent feature of her mental state when she is unwell. (*SAD & Anor v SED* [2017] EWCOP 3, para. 14.)

Dr. F has had the chance to assess DD on three separate occasions since I delivered that judgment on 4 July 2014. His further assessments (report 15 August 2014 and 6 January 2015) have yielded a number of additional insights, namely that DD displays poor reciprocal social interventions, “**poor insight** into her social difficulties”; “stereotyped idiosyncratic us [sic.] of words and phrases” with “clear abnormalities in two way conversations….” (*The Mental Health Trust & Ors v DD & Anor* [2015] EWCOP 4; para 55.)

### Pattern 7

#### Unspecified

In other cases a claim regarding insightlessness appeared *alongside* a claim about incapacity, but without any indication as to how, if at all, the evidence about patient insight was taken to relate to the conclusions drawn about the patient’s capacity. Examples:

CA **did not have capacity or insight** about what was going to happen, or likely to happen… (*Re CA (Natural Delivery or Caesarean Section)* [2016] EWCOP 51, para 26.)

On 15 June 2018, Dr M, a consultant psychiatrist, reported that, in her view, SJ **had no insight**. He was unable to understand or believe information given to him. He was unable to weigh up the pros and cons of treatment. In her view, he lacked capacity to make decisions regarding his health. (*SJ, Re* [2018] EWCOP 28; para 18.)

FT has had dementia since 2012. According Dr V R Badrakalimuthu, a consultant psychiatrist at Parklands Hospital, Basingstoke: “FT has written cheques whilst having no understanding of transactions through his bank account. … **He also has limited insight** into his cognitive impairment and lacks capacity regarding health and welfare.” *FT, Re* [2015] EWCOP 49, para 8

### Pattern 8

#### Interchangeability

In one case (two instances), the concepts of capacity and insight were used in a way that suggested that they were functionally equivalent to one another:

MB exhibits more complete **decision making capacity/insight** when away from NB. (…) It seems to me that this report is evidence of MB’s wishes and feelings in respect of rehabilitation placement being more amenable to suggestion when away from NB, but not of his **decision-making capacity/insight** being any more complete (*London Borough of Brent v NB* [2017] EWCOP 34; para 84).

### Pattern 9

#### Fifth Wheel

In some cases, insightlessness was explicitly included among the grounds for a finding of incapacity, but the other enumerated grounds were sufficient on their own to warrant the same conclusion, with or without the claim about insightlessness. Examples:

The factors that have driven them [specialist registrar and consultant psychiatrist] to that [C lacks capacity to conduct the proceedings and to make decisions about her obstetric care, including whether to undergo a caesarean section] conclusion are[:]

1. C **has no insight** into her current condition or her need for treatment.

2. Her manic symptoms mean she is unable to concentrate for sufficient periods of time to receive all the information relevant to the decisions to be made.

3. If she went into labor the position would be dynamic and require decisions to be made in an ever changing situation, possibly at short notice.

4. Her symptoms prevent her being able to manage these situations as she has limited understanding, limited retention and lacks the ability to weigh the information she has received or use it to make the relevant decisions. (*NHS Trusts v C* (Medical Treatment and Reporting Restrictions Order) (1) [2016] EWCOP 17, para 55)

Dr Andrews provided evidence from his conversations with M during his examination on 28 March to support his conclusions that she lacked capacity in these six areas[:]

1. Deciding on care arrangements – M repeatedly indicated that she was ‘finished with medication at hospital’ and that, if given the choice, would leave. She said she required no support and was now ready to resume independent life. Dr Andrews found that her **insight into her mental health needs** remained significantly impaired. She was unable to attend to, recall or weigh up basic information specific to the role and effects of her new medication… (*AB v HT & Ors* [2018] EWCOP 2, para 35)

### Pattern 10

#### Scope-Delimited Direct Inference

In some cases, insight was held to be essential to capacity for some particular kind of decision, so that a lack of capacity for such a decision could be inferred directly from lack of insight. Examples:

It was decided that he lacked capacity to make decisions about his future residence **because of his lack of insight** into his care needs… (*RD & Ors (Duties and Powers of Relevant Person’s Representatives and Section 39D IMCAS) (Rev 1)* [2016] EWCOP 49, para 20)

Her health and functioning have been maximized but she still does not have capacity to make decisions **which require insight** into her illness (*London Borough of Islington v QR* [2014] EWCOP 26, para 47).

She felt Z over-estimates her ability to keep herself safe, and underestimates her own vulnerability; that in order to be confident that Z has capacity, Dr. Rippon indicated that she would be looking for Z **to develop and display insight**, that she is not putting herself in risky situations and is understanding of other people’s motives… (*Z & Ors, Re* [2016] EWCOP 4, para 49)

### Pattern 11

#### Rejection of Direct Inference

In at least one case (two instances), a direct inference from lack of insight to lack of capacity was rejected:

[T]here is no necessary correlation between **a lack of insight** into schizophrenia and incapacity to decide about surgery [an amputation of her right leg]… (*Heart of England NHS Foundation Trust v JB* [2014] EWCOP 342, para 41)

My conclusion is that JB undoubtedly has a disturbance in the functioning of her mind in the form of paranoid schizophrenia (as **to which she lacks insight**), but that it has not been established that she thereby lacks the capacity to make a decision about surgery for herself. On the contrary, the evidence establishes that she does have capacity to decide whether to undergo an amputation of whatever kind. (*Heart of England NHS Foundation Trust v JB* [2014] EWCOP 342, para 43)

### Pattern 12

#### Indirect Inference

In these cases, lack of insight is invoked as a factor that explains the absence of one or more of the four so-called statutory abilities (to understand, retain, use/weigh, communicate); the absence of the statutory abilities (rather than the absence of insight per se) is then used to justify the conclusion that capacity is absent. Examples:

According to Dr C, he was not orientated in time, although he was to place and person, and his short term memory was significantly impaired. Dr C concluded that PH was not able to weigh up the information concerning his future residence **due to poor insight** into his physical and mental health condition adding to the limitation of his own capabilities and what his care needs entail. Dr C observes: “this lack of insight and understanding of limitations prevented him from balancing the pros and cons remaining at Y Court.” (*PH v A Local Authority* [2011] EWCOP 1704, para 44.)

“**Due to [Miss V]’s lack of insight**, she is unable to understand her vulnerability when out in the community, particularly with regards to strangers approaching her and possibly asking her to get into a car, or the fact that she may get lost and be unable to find her way back. [Miss V] showed no understanding of the support that she needs for personal safety.” (*The Hospital Trust v V & Ors* [2017] EWCOP 20, para 42.)

When Dr P asked GW about her road safety, she was adamant that she never walked in the road. She said that her road sense was excellent. Dr P concluded that it was quite clear that GW **was “quite insightless”** into her lack of road safety and awareness. He concluded that she was unable to understand or weigh up key information relevant to the decision as to whether she should go out of the unit unescorted because she does not understand the risk that her lack of road safety poses. (*GW v A Local Authority & Anor* [2014] EWCOP 20, para 26.)

### Pattern 13

#### Causal Chain

In these cases, which overlap with Pattern 12, insightlessness is explicitly identified as part of a causal chain that starts with a diagnosed disorder. Insightlessness is characterized as an effect of the disorder, with incapacity then characterized as an effect of insightlessness. Example:

In relation to those areas where he is of the opinion that SL lacks capacity, he commented that she simply did not accept the information about her previous condition namely that she had arrived at a state of self-neglect, poor nutrition and weight loss which probably contributed to the development of the ulcers. He considered that the refusal to accept the information **was a result of her lack of insight** into her illness [paranoid schizophrenia], which is itself a result of the illness [8.1.2]. **The natural result of her refusal to accept this information means that she cannot use or weigh it**, although she can retain it because she has no memory problem as such. (*SL, Re* [2017] EWCOP 5, para 9).

## Playing NICE?

There is relatively little formal guidance about the use of evidence concerning insight in the legal assessment of decision-making capacity under the MCA standard. The original MCA Code of Practice, for example, is notably silent on the topic. (In the academic literature, Case 2016 proposes some broad guidance, mainly in the form of a catalog of pitfalls to be avoided.) But in its recently published *Guidelines on Decision-Making and Mental Capacity* (4), the National Institute of Health and Care Excellence (NICE) directed practitioners as follows:

Practitioners should be aware that a person may have decision-making capacity even if they are described as lacking “insight” into their condition. Capacity and insight are 2 distinct concepts. If a practitioner believes a person’s insight/lack of insight is relevant to their assessment of the person’s capacity, they must clearly record what they mean by insight/lack of insight in this context and how they believe it affects/does not affect the person’s capacity. (National Institute for Health and Care Excellence, 2018: 25. Para 1.4.24)

It is important to note that this guidance from NICE was published on 3 October, 2018. Recall that our survey focuses on published CoP judgments from 2007 to 2018; the most recent judgment from our pool of CoP Insight Cases was published on 5 October, 2018. So the judgments we surveyed were prepared and published without the benefit of the NICE guidance. That guidance nonetheless provides a useful standard against which these earlier published judgments can be assessed.

NICE Guideline 1.4.24 effectively includes four discrete pieces of guidance. Specifically, it enjoins practitioners:

- to be alive to the possibility that a person might have decision-making capacity despite being described as lacking insight;
- to be cognizant of the conceptual distinction between insight and capacity;
- to be clear about what they mean by insight or lack of insight; and
- to be clear about how they believe insight or lack of insight affects a person’s capacity.

We were unable to identify any single case among those we surveyed in which all four of these injunctions were squarely satisfied. In the main this was because the concept of insight was so regularly used *without adequate specification as to its meaning*. Our survey also makes clear just how serious a problem this can present. As we have seen, even in the relatively homogenous and formalized discourse of the Court of Protection, the concepts of insight and insightlessness do not have a unitary meaning. A description of P as insightless might mean that P disputes her diagnosis (Pattern 1), or that she does not understand her impairment (Pattern 2); it might mean that she does not recognize risks (Pattern 3) or care/support needs (Pattern 4) or the value of treatment (Pattern 5). And these are by no means the only possibilities. Readers of the rulings are often left guessing as to the meaning of the term, particularly when insightlessness is reported as a ‘bare fact’ (Pattern 6).

As regards the other three NICE injunctions, the record in the COP Insight Cases was mixed. In at least one case the conceptual distinction between insight and capacity seemed to be elided altogether (Pattern 8), but there were also cases which recognized the distinction (e.g., Pattern 12) and one case explicitly noted the possibility of capacity despite insightlessness (Pattern 11), as required by the first injunction.

What about the final NICE injunction—the requirement to be clear about how insight or insightlessness is thought to bear on the question of capacity? This was the point on which we found the broadest spectrum of practice. As we have seen, there were cases where a finding of insightlessness was presented *alongside* testimony regarding a lack of capacity, but without *any* comment on the relation between these two claims (Patterns 7 and 8). In other cases (Patterns 12 and 13), the link between evidence of insight and evidence of capacity was made explicit.

## Limitations and Future Work

As we noted at the outset, this paper is intended as the first in a series in which we seek to answer the call of the *Wessely Review* for more systematic research on the relationship between insight and decision-making capacity. The principal limitations of the present survey derive from its scope and its methodology.

As regards scope, we have confined our attention here to formal judgments of the Court of Protection in England and Wales; even there we have been able to survey only that subset of cases that have been recorded and made publicly available. This is a small subset of the much larger number of cases (both inside and outside the courtroom) in which findings of insight and insightlessness are brought to bear on questions of capacity. We cannot assume that the subset is representative. Moreover, our survey has been based only on the published judgments themselves, including those excerpts from expert testimony that the judges have seen fit to record; we have not had direct access to the expert reports themselves. In future work we will therefore supplement the present survey with material drawn from other sources, both in the UK and internationally.

As regards methodology, our approach here has been almost entirely descriptive. We sought to create a ‘snapshot’ of the current use of evidence concerning insight in the Court of Protection; we have not sought to determine whether that usage is correct. The closest we have come to assessment was in considering the extent to which current CoP usage conforms to NICE Guideline 1.4.24. But we have not sought to assess the adequacy of the NICE Guideline itself. In future work we will need to address the normative question more squarely. On that basis we plan to propose a more fine-grained set of evidence-based guidelines as to whether and how evidence concerning insight and insightlessness should be gathered, presented and adjudicated in the assessment of mental capacity

A final limitation of the present survey pertains to its focus on matters of mental capacity. As we have seen, evidence regarding impaired insight is regularly used in determining whether a person has or lacks mental capacity for a decision that needs to be made. By this legal pathway, insightlessness can become a determinant (and sometimes a decisive determinant) of the scope of a person’s legal capacity and rights. But there is a second legal pathway in which evidence of insightlessness can also be relevant, viz., in decisions about whether to detain a patient for treatment under a Mental Health Act section. In future work we will adapt the method of ‘conceptual geography’ that we have used here in examining the ways in which evidence of insight is used on this second legal pathway, particularly in the context of juridico-clinical decision-making on psychiatric ward rounds.

## Data Availability Statement

The dataset analyzed in this paper is available in the public domain through the British and Irish Legal Information Institute database (BAILII). https://www.bailii.org.

## Author Contributions

The study was designed by WM. The survey and demographic analysis were carried out by SG. The exercise in conceptual geography was undertaken jointly and iteratively by SG and WM. The random sample of all published CoP judgments was carried out by EF. The first draft of the text was prepared by SG with subsequent drafts prepared jointly by SG and WM. All authors contributed to the article and approved the submitted version.

## Funding

Support for the research presented here was provided by the Wellcome Trust as part of its Mental Health and Justice initiative; grant number: 203376/Z/16/Z.

## Conflict of Interest

The authors declare that the research was conducted in the absence of any commercial or financial relationships that could be construed as a potential conflict of interest.
